# LineageSpecificSeqgen: generating sequence data with lineage-specific variation in the proportion of variable sites

**DOI:** 10.1186/1471-2148-8-317

**Published:** 2008-11-21

**Authors:** Liat Shavit Grievink, David Penny, Mike D Hendy, Barbara R Holland

**Affiliations:** 1 The Allan Wilson Centre for Molecular Ecology and Evolution, Massey University, Private Bag 11 222, Palmerston North, New Zealand

## Abstract

**Background:**

Commonly used phylogenetic models assume a homogeneous evolutionary process throughout the tree. It is known that these homogeneous models are often too simplistic, and that with time some properties of the evolutionary process can change (due to selection or drift). In particular, as constraints on sequences evolve, the proportion of variable sites can vary between lineages. This affects the ability of phylogenetic methods to correctly estimate phylogenetic trees, especially for long timescales. To date there is no phylogenetic model that allows for change in the proportion of variable sites, and the degree to which this affects phylogenetic reconstruction is unknown.

**Results:**

We present LineageSpecificSeqgen, an extension to the seq-gen program that allows generation of sequences with both changes in the proportion of variable sites and changes in the rate at which sites switch between being variable and invariable. In contrast to seq-gen and its derivatives to date, we interpret branch lengths as the mean number of substitutions per **variable** site, as opposed to the mean number of substitutions per site (which is averaged over all sites, including invariable sites). This allows specification of the substitution rates of variable sites, independently of the proportion of invariable sites.

**Conclusion:**

LineageSpecificSeqgen allows simulation of DNA and amino acid sequence alignments under a lineage-specific evolutionary process. The program can be used to test current models of evolution on sequences that have undergone lineage-specific evolution. It facilitates the development of both new methods to identify such processes in real data, and means to account for such processes. The program is available at: http://awcmee.massey.ac.nz/downloads.htm.

## Background

Simulated sequence data are widely used for hypothesis testing [1], for evaluation of phylogenetic methods under different parameter settings [2-4], for testing the effect of model misspecification on tree reconstruction [5-7], for development of new models and methods [8,9], and for approximate Bayesian inference [10]. For these applications, it is important that the processes used to produce the simulated data closely model the underlying biological processes. Commonly used phylogenetic sequence generators employ homogeneous, time reversible, stationary models of molecular sequence evolution. These phylogenetic models assume that the overall rate of substitution is the only parameter that may change along the tree and do not allow changes in other parameters, such as the rate matrix, the distribution of rates across sites and the proportion of variable sites.

It is known, however, that as sequences diverge they can acquire independent properties. In particular, the proportion of variable sites can evolve in a lineage-specific manner due to changes in evolutionary constraints [11,12]. The proportion of variable sites in a lineage will affect its estimated substitution rate [13]. Failure to account for changes in the proportion of variable sites can result in erroneous rate estimates that may affect tree estimation [5,14]. Indeed, change in the proportion of variable sites is thought to be one of the main causes of long-branch attraction [12,15].

In addition to the possible shift in the proportion of variable sites, it is known that sites can switch between variable and invariable states due to drift. Note that **invariable** sites (which we are concerned with in this paper) are sites for which the probability of character substitution is zero; as opposed to **invariant** sites for which the probability of character substitution is greater than zero but for a certain group (sample) of taxa no substitution is found. The strict covarion model [16] allows sites to switch between variable and invariable states; however, at equilibrium the proportion of variable sites is constant over the different lineages. Several extensions of the covarion model [17-19] are implemented in the sequence generator seq-gen-aminocov [19]. However, to date, there is no model that allows for change in the proportion of variable sites.

Using partitions (a partition is group of consecutive sites that are simulated on the same underlying tree), lineage-specific proportions of variable sites have been simulated with sequence generators such as seq-gen [20], seq-gen-cov [21], and seq-gen-aminocov [19]. These simulations have proven to be very useful in facilitating our understanding of the process of lineage-specific evolution. However, the use of these programs for the purpose of simulating changes in the proportion of variable sites is limited to trees with very few 'events', where an event is defined as a position on the tree where a change in the process of evolution occurs, e.g. a change in the proportion of variable sites. This is because different proportions of variable sites are generated using pre-defined partitions, where each partition is simulated on a tree with different branch lengths (zero branch lengths are used for invariable sites). For two events, in which the proportion of variable sites changes, there are 8 partitions [5]. In general there are 2^(1+number of events) ^partitions (M. Steel, personal communication), so creating the input for such simulations becomes a difficult task. Furthermore, in seq-gen, invariable sites can be incorporated into sequences by either simulating on different partitions (where a partition for invariable sites is simulated on a zero length tree), or specifying a proportion of invariable sites (Pinv) using the -i option. Intuitively, one might expect the processes of evolution simulated by these two methods to be equivalent, but this is not the case. In seq-gen and its modifications published to date, branch lengths are defined as the mean expected number of substitutions per site. When sequences are simulated with a specified proportion of invariable sites, the branch lengths specified by the user are rescaled (increased) by the program to compensate for the proportion of invariable sites. Hence, increasing the proportion of invariable sites (for which the substitution rate is zero) forces a greater substitution rate on the variable sites. For example, with 80% invariable sites and an expected mean number of substitutions of 0.02, the mean number of substitutions of the variable sites will be rescaled to 0.1. Although this branch rescaling is consistent with the definition of branch lengths as the mean expected number of substitutions per site, we found that many researchers are not aware of it. Moreover, using partitions does not allow changes in the on/off switch rate of the covarion model. We have developed a program that allows the user to simulate sequence data containing changes in the proportion of variable sites, and changes in the covarion switch rate, without the need to specify partitions or rescale branch lengths.

## Implementation

**Figure 1:**
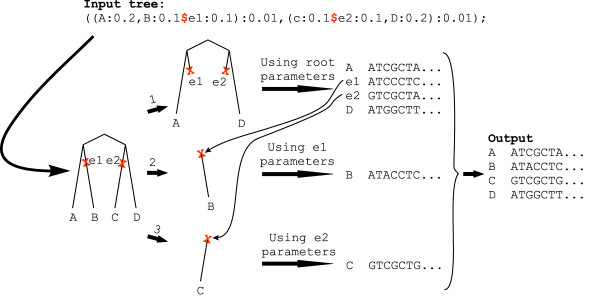
**An example workflow of LineageSpecificSeqgen**. For each tree, the program creates a random root sequence according to the parameters specified. The program then evolves the sequences, according to the parameters given for the root, along the subtree beneath the root (excluding parts of the tree that are beneath events). The resulting sequence at each event is then used as an ancestral sequence for the subtree beneath that event, and the sequences are evolved along that subtree according to the parameters specified for that event. The output is an alignment of the resulting sequences at the tips.

LineageSpecificSeqgen is a command-line controlled program written in C. The program uses, as much as possible, the code from seq-gen and its derivatives [19-21]. Given a rooted tree, specified events (in which changes in the process occur), and a set of parameters, the program generates a user-specified number of datasets (nucleotide or amino-acid). An example workflow of the program is illustrated in Figure 1. The input is two text files – a tree file and a parameter file. The tree file contains one or more trees in a format which is based on the Newick format. Events on the tree are marked using a $ sign and are given names. Lengths are specified for all branches of the tree; for branches with events, the length before and the length after the event must both be specified. The parameter file contains the changes in the proportion of variable sites, and/or the switch rate of the covarion process, at each event. Any number of events can be specified. A change in the proportion of variable sites is specified using two parameters; the proportion of sites that were invariable and became variable at the event, and the proportion of sites that were variable and became invariable at the event.

For each input tree, the program will generate *n *subtrees (*n *= 1+number of events), with each event on the tree defining a cutting point (see Figure 1). For each input tree and each dataset, sequences are first simulated on the subtree under the root and then on the subtree under each event in an iterative manner. An array holding the state (variable/invariable) of each position is updated at each event according to the change in the proportion of variable sites specified by the user. Events can be specified as correlated, although by default they are non-correlated. For correlated events the positions of sites that switch state are identical, for non-correlated events these positions are independent. An array holding the hidden states of the covarion model is also passed down the tree. For each site, along each branch, exponential times for switches are generated; the hidden states array is updated at the internal nodes of each subtree according to the specified covarion model and the switch rate for each event. The sequence at each event is used as the ancestral sequence for the subtree beneath it. The output is an alignment of the resulting tip sequences.

For the reasons described in the Background, we added a default option where branch lengths are defined as the mean expected numbers of substitutions per variable site. This definition allows the substitution rate across variable sites to be independent of the proportion of invariable sites. When branch lengths are defined as the mean expected numbers of substitutions per variable site, the processes of evolution simulated by both specified partitions and specified Pinv are equivalent.

## Results and discussion

### Example 1 – generating data containing a change in the proportion of variable sites

**Figure 2:**
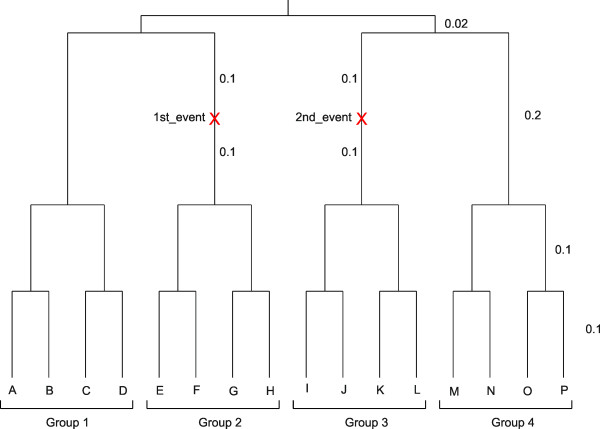
**16-taxon rooted balanced tree used for simulation**. Sequences were generated on a 16-taxon rooted balanced tree. The tree is comprised of four groups of four taxa each. There are two correlated events on the tree in which the proportion of variable sites changes. The events are located on the two non-sister lineages 2 and 3.

To demonstrate the use of the program for generating datasets containing a change in the proportion of variable sites, the Jukes-Cantor (JC) model was used to generate sequences of length 10,000 bp on the 16-taxon rooted balanced tree shown in Figure 2. This tree is input as:

((((A:0.1, B:0.1):0.1,(C:0.1, D:0.1):0.1):0.2,((E:0.1, F:0.1):0.1,(G:0.1, H:0.1):0.1):0.1$1st_event:0.1):0.02,(((I:0.1, J:0.1):0.1,(K:0.1, L:0.1):0.1):0.1$2nd_event:0.1,((M:0.1, N:0. 1):0.1,(O:0.1, P:0.1):0.1):0.2):0.02);

In this example, the proportion of invariable sites (Pinv) at the root was set to 0.8. At each of the two events 0.2 of the invariable sites were "switched on" (became variable). The two events were set to be correlated so that the positions of sites that are turned on in the two events are identical. The expected proportion of variable sites in groups 1 and 4 is thus 0.2, and the expected proportion of variable sites in groups 2 and 3 is 0.36. Consequentially, 0.64 of the sites are invariable across all four groups, and 0.16 of the sites are variable in groups 2 and 3 and invariable in groups 1 and 4. For comparison, two control datasets were generated on a 16-taxon rooted balanced tree without the two events; the same branch lengths were used as before, and Pinv was set to either 0 or 0.8. For each group, and each pair of groups, the number of sites that varied in each of the three simulated sequence alignments is shown in Table 1.

**Table 1:** Number of sites that vary in each of the four groups, and each pair of groups, for the three datasets.

Group/s	No events Pinv = 0 (all sites are variable)	No events P_inv _= 0.8	P_inv _= 0.8 Two correlated events Pvar^+ ^= 0.2
1	4509	905	911
2	4363	922	1574
3	4347	925	1635
4	4410	913	919
1 and 2	1947	404	375
1 and 3	1930	410	416
1 and 4	2003	411	417
2 and 3	1915	426	709
2 and 4	1901	431	409
3 and 4	1898	421	408

### Example 2 – testing tree reconstruction accuracy for data containing a change in the proportion of variable sites

**Figure 3:**
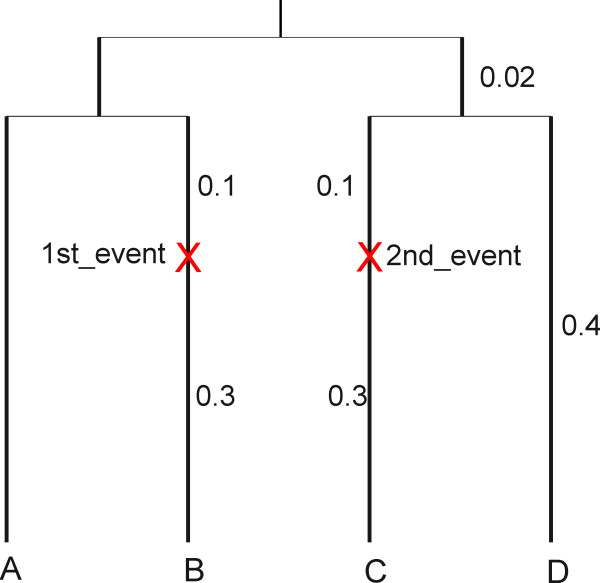
**4-taxon rooted balanced tree used for simulation**. Sequences were generated on a 4-taxon rooted balanced tree. There are two correlated events on the tree in which the proportion of variable sites changes. The events are located on the two non-sister lineages 2 and 3.

To demonstrate the use of the program for testing tree reconstruction accuracy for datasets containing a change in the proportion of variable sites, the JC model was used to generate 100 datasets of length 10,000 bp on the 4-taxon rooted balanced tree shown in Figure 3.

**Figure 4:**
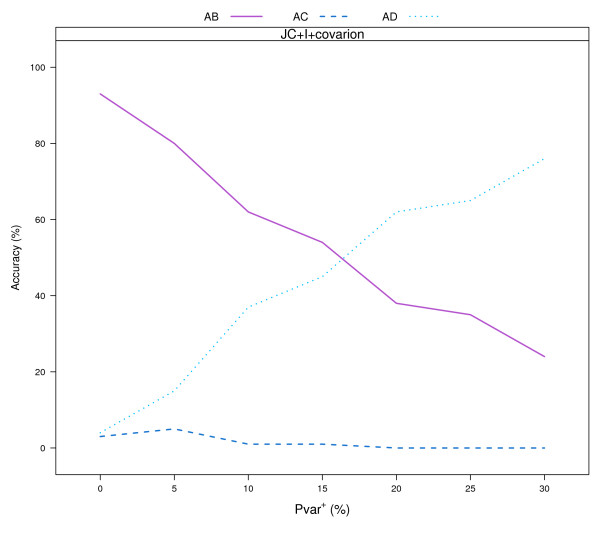
**Tree reconstruction accuracy for the simulated 4-taxon datasets, using JC+I+covarion model**. The number of times with which each of the three possible 4-taxon trees was reconstructed, with the highest proportional frequency in the Bayesian analysis, assuming JC+I+covarion model. The higher the increase in the proportion of variable sites in lineages B and C, the lower the tree reconstruction accuracy.

This tree is input as:

((A:0.4, B:0.3$1st_event:0.1):0.02,(C:0.3$2nd_event:0.1, D:0.4):0.02);

As in the former example, Pinv at the root was set to 0.8 and the two events were set to be correlated. At each of the two events Pvar^+ ^= (0, 5, 10, 15, 20, 25, 30) percent of the invariable sites were "switched on". The program MrBayes [22] was used to reconstruct the trees, assuming a JC model with invariable sites and a covarion process (JC+I+covarion). The number of times with which each of the three possible 4-taxon trees was reconstructed with the highest proportional frequency in the Bayesian analysis were compared to determine tree reconstruction accuracy. As shown in figure 4, the higher the increase in the proportion of variable sites in lineages B and C, the lower the tree reconstruction accuracy. These results suggest that, at least for some parts of the parameter space, a covarion model which assumes a constant proportion of variable sites is not adequate for tree reconstruction from data containing changes in the proportion of variable sites.

## Conclusion

LineageSpecificSeqgen is a sequence generator that allows simulation of changes in the proportion of variable sites, a biochemically realistic process of evolution. It is useful for testing current models of evolution on sequences that have undergone lineage-specific evolution, developing methods to identify such processes in real data, and developing means to account for such processes.

## Availability and requirements

• Project name: LineageSpecificSeqgen

• LineageSpecificSeqgen, including the source code and documentation, can be downloaded from http://awcmee.massey.ac.nz/downloads.htm.

• Operating System: The program can be compiled and run on Unix, Linux, and Mac OS.

• Programming Language: ANSI C.

• Other requirements: None.

• License: GNU GPL.

• Any restrictions to use by non-academics: None.

• LineageSpecificSeqgen is provided with no guarantee or warranty of any kind, although the authors are happy to provide assistance if needed.

## Authors' contributions

LSG has developed and implemented the code for the program, and had written the first draft of this manuscript. DP, MDH and BRH supervised the project. All the authors contributed to the writing of this manuscript.
