# LineageSpecificSeqgen: generating sequence data with lineage-specific variation in the proportion of variable sites

**DOI:** 10.1186/1471-2148-9-200

**Published:** 2009-08-13

**Authors:** Liat Shavit Grievink, David Penny, Mike D Hendy, Barbara R Holland

**Affiliations:** 1 The Allan Wilson Centre for Molecular Ecology and Evolution, Massey University, Private Bag 11 222, Palmerston North, New Zealand

## Abstract

Correction to Shavit Grievink L, Penny D, Hendy MD, Holland BR: **LineageSpecificSeqgen: generating sequence data with lineage-specific variation in the proportion of variable sites**. *BMC Evol Biol *2008, **8**(1):317.

## Correction

Since publication of our article [1], we discovered an error in the second example. For this example, we state in the paper, we used the program MrBayes [2] with the JC+I+Cov model. However, we now found that, albeit appearances, this model is not implemented in MrBayes [2]. In fact, no combination of I+Cov (e.g. HKY+I+Cov, GTR+G+I+Cov) is currently implemented in MrBayes [2]. Instead, the program ignores the I parameter, so tree reconstruction in this example was therefore effectively done using the JC+Cov model. This does not affect the conclusion of our paper that phylogenetic estimation can be misleading for sequence data simulated with lineage-specific properties.
